# Supplementary material for: Prevalence of asymptomatic malaria infection by microscopy and its determinants among residents of Ido-Ekiti, Southwestern Nigeria
Source: PLoS One. 2023 Feb 14;18(2):e0280981. doi: 10.1371/journal.pone.0280981 (PMC9928065; doi:10.1371/journal.pone.0280981)
Supplement: S1 File — (PDF) [file pone.0280981.s001.pdf]

DRYAD

Title: Prevalence Of Asymptomatic Malaria Infection By Microscopy And Its Determinants Among Residents of Ido-Ekiti, Southwestern Nigeria

Bello, Ibrahim Sebutu, Obafemi Awolowo University Teaching Hospital Complex**,** https://orcid.org/0000-0002-5326-2894

IBRAHIM, Azeez Oyemomi, Federal Teaching Hospital, Ido-Ekiti, Ekiti State, Nigeria

AJETUNMOBI, Adewumi Oluwaserimi, Federal Teaching Hospital, Ido-Ekiti, Ekiti State, Nigeria

AYODAPO, Abayomi, University College Hospital, Ibadan. Nigeria

AFOLABI, Babatunde Adeola, Ladoke Akintola University Teaching Hospital, Oshogbo, Osun State, Nigeria

ADENIYI, Makinde Adebayo, Federal Medical Centre, Abeokuta, Ogun State, Nigeria.

bello.ibrahim@gmail.com

Publication date: Not available

Publisher: Dryad

Research Facility: Federal Teaching Hospital, Ido Ekiti*

https://doi.org/10.5061/dryad.gqnk98ssx

Citation

Bello, Ibrahim Sebutu et al. (2023), Title: Prevalence Of Asymptomatic Malaria Infection By Microscopy And Its Determinants Among Residents of Ido-Ekiti, Southwestern Nigeria , Dryad, Dataset, https://doi.org/10.5061/dryad.gqnk98ssx

Abstract

Background: Asymptomatic malaria infections have received less attention than symptomatic malaria infections in major studies. Few epidemiological studies on asymptomatic malaria infections have often focused on pregnant women and children under-five years of age as the most vulnerable groups. However, there is limitation on data regarding asymptomatic infections among the old adult populations, particularly in the study area. Therefore, this study determined the prevalence of asymptomatic malaria infection by microscopy and its determinants among residents of Ido- Ekiti, Southwestern Nigeria.

Methods: A hospital-based cross-sectional study was conducted between July and September 2021 among 232 consenting apparently healthy individuals aged 40 years and above who were recruited during a free health screening program using a standardised interviewer-administered questionnaire. The questionnaire sought information on respondents' socio-demographics, presence and types of co-morbidity, and the prevention methods being adopted against malaria infection. Venous blood samples were collected and processed for asymptomatic infections using Giemsa-stained blood smear microscopy. Data were analysed using SPSS version 21. Multivariate logistic regression was used to identify factors associated with asymptomatic infections.

Results: Of the total 232 respondents, 19.0% (48/232) were confirmed to be infected with *Plasmodium falciparum* (95% confidence interval (CI): 14.1% - 24.6%). Lack of formal education (Adjusted odds ratio (AOR): 5.298, 95% (CI): 2.184-13.997), being diabetic (AOR: 4.681, 95% CI: 1.669-16.105), and not sleeping under Long Lasting Insecticide Net (LLINs) (AOR: 4.594, 95% CI: 1.194-14.091), were the determinants of asymptomatic *Plasmodium falciparum* infection.

Conclusion: The prevalence of asymptomatic *Plasmodium falciparum* was 19%. Lack of formal education, being diabetic, and not sleeping under LLINs were the determinants of asymptomatic infections.

Methods

Data collection instruments: The two instruments for data collection were the standardised interviewer-administered questionnaire and the data collection form. The questionnaire sought information about the respondents' socio-demographic characteristics (such as age, gender, education, occupation, and location), mode of malaria prevention adopted by them, and their past medical history. They were also assessed for the presence of co-morbid conditions such as hypertension, diabetes mellitus, Human Immunodeficiency Virus/ Acquired Immune Deficiency Syndrome (HIV/AIDS), Chronic Obstructive Pulmonary Disease (COPD) and Heart failure. These were self-reported.

Clinical parameters of the respondents:

Microscopy for asymptomatic infections: Capillary blood samples were collected by finger pricking using a disposable lancet. The thick and thin films were made from the blood sample. The thick and thin smears were prepared on clean, dry microscope glass slides and were allowed to dry. The thin smear was

fixed in methanol, and both smears were stained with 5% Giemsa. The stained slides were taken to the hospital laboratory, where parasitological examinations were made independently by two malaria microscopists, with discrepancies resolved by a senior microscopist who ensured quality control. A slide was declared negative if parasites were absent after examining 200 high-power fields. Parasite density was quantified against 200 leucocytes on an assumed leucocyte count of 8000 per ul of blood [17]. The degree of parasite density was graded as mild, moderate, and severe when the counts were between 1-999 parasites/ul, 1000-9999/ul, and > 10,000/ul, respectively, following the method described elsewhere [18].

Parasites/ul = No. of asexual stages x 8000 leukocytes/200 leukocytes

Determination of Packed cell volume (PCV): A micro-haematocrit tube was filled with blood and centrifuged in a micro-haematocrit rotor at 10,000 rpm for 5 minutes. PCV was read using the micro-haematocrit reader and recorded as no anaemia (PCV≥30%), mild anaemia (25-29%), moderate anaemia (20-24%), and severe anaemia (<20%) [19].

Statistical analysis: Data were coded, cleaned, entered, and analysed using IBM SPSS for window version 21.0 (IBM Corp., Armonk, NY, USA), respectively. Quantitative data were expressed as mean ± standard deviation. Frequencies were used to determine the respondents' prevalence of asymptomatic *Plasmodium* infection. Binary logistic regression was employed to assess the determinants of asymptomatic *Plasmodium* infection. Variables significant at P-value < 0.05 in the univariate logistic regression were selected for multivariate logistic regression analysis model. Odds ratios with 95% confidence intervals were calculated and P-value< 0.05 was considered statistically significant.

Usage Notes

Microsoft Excel

Reviewer URL

You can share this version of your dataset files with others using the url below.

Click the *Select Text* button and then copy and paste the URL.

Sharing Link

https://datadryad.org/stash/share/EuWsEPGXCoOg02tC-3_FnpBjvfBL4OzWUemI Select Text

Funding

None, Award: Not applicable

Data files

Download dataset

January 25, 2023

** changes not displayed to the public*

Metrics

0 views

0 downloads

0 citations

Keywords

Clinical medicine

Determinants

Ido- Ekiti

Keywords: Asymptomatic malaria infections

residents

rural Nigeria.

License

This work is licensed under a CC0 1.0 Universal (CC0 1.0) Public Domain Dedication license.

PUBLIC DOMAIN

Contact us Follow us on Twitter Check out our blog

Copyright (c) 2023 Dryad
