# Supplementary material for: Prevalence of asymptomatic malaria infection by microscopy and its determinants among residents of Ido-Ekiti, Southwestern Nigeria
Source: PLoS One. 2023 Feb 14;18(2):e0280981. doi: 10.1371/journal.pone.0280981 (PMC9928065; doi:10.1371/journal.pone.0280981)
Supplement: Attachment — Submitted filename: Review comments.docx [file pone.0280981.s003.docx]

**Socio-Demographic Profiles, Co-Morbid Conditions and Preventive Measures as**

**Determinants of Asymptomatic Malaria Infections Among Older Age Population in**

**Rural Southwest Nigeria**

**Review Comments**

General: The aim was to assess the prevalence and determinants of asymptomatic infections among individuals 40 years of age and above, resident in a rural community of Ekiti State, Nigeria. The study attempts to present useful information regarding malaria among the elderly, however the definition of ASM may be seriously flawed, given that the study population comprised of individuals visiting a hospital due to illness and with malaria parasites. Thus, the study population can be classified as having clinical malaria and not asymptomatic infection. The interpretations of the statistical analyses are inaccurate. Revision of the language to improve readability, particularly of the introduction sections, using a copy-editing tool, is recommended.

The following minor and major corrections/suggestions could help improve the manuscript:

**Title**: The authors may consider putting the name of the rural community in the title. The title should indicate that asymptomatic infections were determined by microscopy

**Abstract**: Overall, there is coherence in abstract but could be improved.

Line 31 to 34: Authors should consider restructuring the sentence to improve readability.

Methods: Authors should consider inclusion of the year of the cross-sectional sampling and the name of the rural community where samples were drawn, in the abstract.

Conclusion line 43: The ‘arguments for high prevalence of asymptomatic malaria in rural settings’, are not shown in results, so the sentence should be clarified.

**Introduction**: Authors should consider revising the language to improve readability, using a copy-editing tool.

Line 49 to 51: The presence of malaria parasitaemia without symptoms of illness may best be described as an asymptomatic infection, so authors should consider replacing asymptomatic malaria with asymptomatic (malaria) infection.

Line 75 to 78: There are many old-age-associated diseases with socio-economic implications, and planning an effective malaria control programme is not entirely dependent on knowing the prevalence of asymptomatic infections among the elderly. The highest burden of malaria is among children under five years of age, who are the target of several interventional programmes, so the rationale for the study is weak.

**Materials and methods:**

Line 87: Authors should provide the names of the study institution and the rural community.

Line 97 to 99: Authors should note that an infection identified during a hospital visit will not qualify as asymptomatic.

Line 100: Authors should specify ‘recent history’ (eg. 24 or 48 hours ago or a week ago etc.) in their inclusion criteria/definition of asymptomatic infection

Line 104: Why did Authors exclude individuals with mental illness? Was axillary temperature ≥ 37°C also excluded?

Line 110 to 120: The procedure of the systematic sampling is impressive, however, it is unclear why the sampling is considered random, as the selection of the first respondent appears systematic.

Line 147: May not be relevant.

Line 148, 157: Authors should structure as a minor heading, removing the indent.

Line 161 to 163: Authors should ensure that the referenced treatment guidelines are for the asymptomatic infections (study target population) and not management of clinical malaria.

Authors should provide a clear definition of asymptomatic infection in the methods section.

**Results** The authors aim to demonstrate association between several predictor variables and asymptomatic infections, however, the lack of a clear definition for asymptomatic infection (ASM) detracts from the integrity of the data collected. Specifically,

Line 176: Of respondents who tested positive for parasitaemia, how many had symptoms?

Line 179, Table 1: The proportion of individuals testing negative has not been shown to have asymptomatic infections (by PCR) and so should be removed from Table 1, or the heading re-worded. The individuals testing negative should not be placed under the heading ‘Asymptomatic Malaria Parasitaemia’ in the table.

Line 183 to 203: Table 2, 3 and 4 show the distribution of **microscopy** positive and negative tests by age, sex, occupation etc… and whether the overall differences in the distribution are significant. The tables do not show association between the predictor variables and asymptomatic infections (ASM). Authors should present their results/data properly, for statistical accuracy.

Line 188, Table 2: The reference variables are unclear, if association between the predictors and ASM is the goal. The negative population may or may not have malaria parasites by PCR, so ‘**Malaria’** should be replaced with the word ‘**Microscopy’**.

Line 216, Table 6: The response variable (malaria test positive with or without symptoms = ASM?) for which the regression analyses was performed is missing from the table, so it is difficult to understand how the results were interpreted by the authors.

**Discussion**:

The discussion reads well, based on the results presented. However, due to the lack of a clear definition for ASM and inaccurate interpretations of the statistical analyses, the discussions and conclusions cannot be scientifically supported. The discussion should be re-written after the corrections in the other sections.
