# Supplementary material for: Prevalence of asymptomatic malaria infection by microscopy and its determinants among residents of Ido-Ekiti, Southwestern Nigeria
Source: PLoS One. 2023 Feb 14;18(2):e0280981. doi: 10.1371/journal.pone.0280981 (PMC9928065; doi:10.1371/journal.pone.0280981)
Supplement: Attachment — Submitted filename: Response to Reviewers_1.doc [file pone.0280981.s004.doc]

**PLoS ONE Summary of Reviewers’ comments and Author’s response**

**Dear Editorial team/Reviewers For PONE-D-22-24029**

**Thank you very much for the efforts and input in reviewing our manuscript. Your contributions to improving our manuscript are well noted and appreciated. Please find attached our responses to your comments.**

| **S/N** | **Editorial comments** | **Author’s response** |
| --- | --- | --- |
| 1 | Please ensure that your manuscript meets PLoS ONE's style requirements, including those for file naming. The PLoS ONE style templates can be found at | We have studied the style requirements which we have applied in our responses |
| 2 | Please amend your current ethics statement in the Materials and Methods section of your manuscript to include the full name of the ethics committee/institutional review board(s) that approved your specific study. | This has been done. Please check line 163-164 |
| 3 | We note that you have stated that you will provide repository information for your data at acceptance. Should your manuscript be accepted for publication, we will hold it until you provide the relevant accession numbers or DOIs necessary to access your data. If you wish to make changes to your Data Availability statement, please describe these changes in your cover letter and we will update your Data Availability statement to reflect the information you provide. | Our data availability statement has been described in our new cover letter. (Check the cover letter). |
|  | Additional Editor Comments:  After careful consideration, we felt that your manuscript requires revision, following which it can possibly be reconsidered. Although your manuscript was of interest to the reviewer, major concerns were related to study design and results. As stated by the reviewers the definition of ASM may be seriously flawed, given that the study population comprised of individuals visiting a hospital due to illness and with malaria parasites. The interpretations of the statistical analyses also need to be revised. In addition, a significant number of issues should be clarified and/or adjust otherwise the MS’s results may be compromised. Finally, we strongly suggest that the MS should be submitted to a copy-editing process. For your guidance, a copy of the reviewers' comments was included below. | All the issues raised here have been addressed in the corrected manuscript. |

| S/N | Reviewers’ comments | Author’s response |
| --- | --- | --- |
| #1.1 | Reviewer #1:  1. Title needs modification. Because, it does not much with your objective. So, I recommend your title to be modified as’’ Prevalence of asymptomatic malaria infection and its determinants among older aged patients attending a tertiary hospital in rural Southwestern Nigeria. In addition, what does older aged mean? You have to give clear definition in your operational definition section.  2. In line number 5, you have to write the Authors’ name with superscript first and write their affiliations in increasing order using super scripts. You can give similar super script number for authors at the same affiliation and separate corresponding Author with star mark from other authors. So, remove email, OR Cid etc from each. You have to also write all the abbreviations in full terms here.  Lines24 – 27 write as: *Correspondence author: Ibrahim Sebutu BELLO  Email: [bello.ibrahim@gmail.com](mailto:bello.ibrahim@gmail.com). This one is enough and remove other sentences  3. Abstract:  Here, there is no background section. You have to write the background with statement of the problem in short and show clear message before putting objective. The objective remains true only if you modify your title as I mentioned earlier.  4. Methods: Rewrite lines 31-34 clearly including study period, kind of cross sectional design whether community based, health facility based, school based etc.  5. Results: line 39-40, write “being diabetes” as “being diabetic”  6. Conclusion:  Lines 44-45 “The findings may guide ….” is not vital here. But, you have to describe the key findings, for example, prevalence, species and key determinants/factors/ associated.  Introduction  Line49: ref [1] you used is not about ASM. But, it is about worldwide prevalence of anemia. Even it is too old. Please use appropriate citation and remove this.  Methods and materials  Line87: Write Study design area and period as subtitle/ heading2/, then, write like” A hospital based descriptive cross sectional study was carried out at… from…to … (i.e. period)  What is descriptive cross sectional study design mean?  Line89: the census data (2006) is too old. Can’t you find current/ updated/ data?  Remove line 97. Since, I have mentioned it to write at the beginning of method section.  Line 100: why do these older aged (despite its definition in need?) persons came to hospital? Since they area symptomatic and your study also is on asymptomatic cases. People with no signs/symptoms do not attend health institutions. So, how did you get your study subjects in tertiary hospital?  In addition, elderly people especially those above 60 usually remain symptomatic if bitten as they have suppressed immune system. So, how can they remain asymptomatic and be your study subjects. That is why I asked you to operationalize “older aged patients” at the very beginning. I want to hear clear cut of age you used in your study.  Line 104: Also, those …anti-malarial (put treatment) ….  Line 106: sample size… can you clearly show us how you calculated sample size? Here, 16.8% prevalence you used is that obtained by PCR in previous study. So, why did you use PCR value rather than using the value obtained by Microscopy (i.e., 7.2%) since the method you conducted to detect ASM is microscopy.  Line 120: remove “The study was conducted between July and October 2021” as I informed you to write in “Study design, area and period section”.  Line 148 – 149: What about to determine P. vivax and other species? Reference [18] is placed in two areas; lines 143-144 and line 156, which are very far and different ideas. Please write appropriate citation for these ideas each.  Lines 162- 165: According to the national malaria drug policy, …. These sentences are the same and identical with the sentence in lines 143-145, but, with different references. Please see it again and remove either of.  Results  Please describe the species detected!  Line 176: write as “in all, only 19% (44/232)…”  Table1: what were your criteria to classify parasitemia as mild, moderate and severe?  Line 182-184, do you mean the association is in binary logistic regression?  Line 185-187: how do you see the positivity rate of ASM in age groups of 61-80 years old, farmers and informal27/09/2022, 04:10  Education? Is that the reality? They have to be symptomatic rather than asymptomatic carriers especially those in 61-80 yrs.  Table 2: in your income classification, is 1.9 dollars daily/weekly/monthly income? How did you take 1.9 dollar as baseline to classify income? Why not 2, 3…or other dollars?  Line 191-193: Do you think that co morbidity does not worsen the signs/symptoms of malaria? For example, why do people with diabetes remain asymptomatic for malaria than non-diabetic?  Line 205: what is window net? Is it physical or chemical barrier? How is it applied?  Table 6: here you used Odds ratio as measure of association. But, you applied chi-square in the above. Why do you need to apply two different measures of association? Why couldn’t you use either of one in all?  Discussion  Line 226: put CI in prevalence. B/c the reader can easily understand and compare your value with the values of other studies.  Line 229: reference [21] does not much with the data you used to compare your result. Please see it again.  Line 235-236: However, other studies have disagreed with this report [23, 24]. What was the report of other studies here? You have to specify so that the readers can easily understand. By the way, reference 23 is not relevant and not used to compare your study which is ASM. But, that one is done from febrile/symptomatic/ patients and hence you have to remove.  Line 240: ref [26] can’t be used to compare your study. B/c that study ws among children while yours is among older aged. Please note that; in discussion, you have to use the data from the studies among similar study subjects, similar methodology, similar disease condition/asymptomatic only/ etc.  Line 254: …but opposite to several other studies [14, 27]. What do you mean by opposite? Is that lower/ higher? Also please specify the number when you say consistent, higher/ lower in each section so as to make clear for the readers.  Line 259: This may be further aggravated by the low immunity of urban dwellers. Why is the immunity of urban dwellers low?  Line267- 275: In this study, the respondents with mild and moderate anaemia…  This is not your concern and is out of your objective and hence no need to discuss about. Remove it.  Acknowledgement: if this section is needed, why don’t you acknowledge your study participants? They are everything for your study.  General comment: Italicize the genus and species names of parasites/ microorganisms in every section. | The title has been modified (check Line2-3)  Age classification has been modified in line with WHO group. (check inclusion criteria and table 1)  This has been addressed in the authors’ affiliation (check 5-14)  Correspondence author:  This has been addressed (check Line 15)  3. Abstract:  Background has been included. The objectives have been modified (check L23-25)  4. Methods: This has been addressed(L28-30)  5. Results: This has been addressed (L39)  6. Conclusion: This has been modified (L43-45)  Introduction  Ref [1] has been replaced with appropriate citation (L330, Reference 1)  Methods and materials  All the issues raised here have been addressed under methods and materials. (L87-113)  This has been addressed (L104-106 & 108-109)  Clear explanation on sample size calculation has been provided. (L114-119)  This has been addressed  One of these statements has been expunged and appropriate citation has been included.  This has been addressed (L172-173)  The species detected has been described (L207)  This has been addressed  (L206)  The criteria to classify parasite density has been included under methods and material and was referenced as [18].(L153-155)  This has been addressed  This has been addressed  (Table1& Line 202-203)  This has been addressed  (L281-294)  This has been addressed (L182-183)  Table 6 has been modified (Table 6, L250)  Discussion  CI has been added (L258)  Ref 21 has been replaced  The whole discussion has been re-written.  (L261-304)  The whole discussion has been re-written.  (L261-304)  It has been removed  Acknowledgement:  The participants have been acknowledged (L326-327)  General comment: The species and genus have been italicized throughout in the manuscript. |
|  | Reviewer #2: Socio-Demographic Profiles, Co-Morbid Conditions and Preventive Measures as Determinants of Asymptomatic Malaria Infections Among Older Age Population in Rural Southwest Nigeria Review  Comments General: The aim was to assess the prevalence and determinants of asymptomatic infections among individuals 40years of age and above, resident in a rural community of Ekiti State, Nigeria. The study attempts to present useful information regarding malaria among the elderly, however the definition of ASM may be seriously flawed, given that the study population comprised of individuals visiting a hospital due to illness and with malaria parasites. Thus, the study population can be classified as having clinical malaria and not asymptomatic infection. The interpretations of the statistical analyses are inaccurate. Revision of the language to improve readability, particularly of the introduction sections, using a copy-editing tool, is recommended.  The following minor and major corrections/suggestions could help improve the manuscript:  Title: The authors may consider putting the name of the rural community in the title. The title should indicate that asymptomatic infections were determined by microscopy  Abstract: Overall, there is coherence in abstract but could be improved.  Line 31 to 34: Authors should consider restructuring the sentence to improve readability.  Methods: Authors should consider inclusion of the year of the cross-sectional sampling and the name of the rural community where samples were drawn, in the abstract.  Conclusion line 43: The ‘arguments for high prevalence of asymptomatic malaria in rural settings’, are not shown in results, so the sentence should be clarified.  Introduction: Authors should consider revising the language to improve readability, using a copy-editing tool.  Line 49 to 51: The presence of malaria parasitaemia without symptoms of illness may best be described as an asymptomatic infection, so authors should consider replacing asymptomatic malaria with asymptomatic (malaria) infection.  Line 75 to 78: There are many old-age-associated diseases with socio-economic implications, and planning an effective malaria control programme is not entirely dependent on knowing the prevalence of asymptomatic infections among the elderly. The highest burden of malaria is among children under five years of age, who are the target of several interventional programmes, so the rationale for the study is weak. | Reviewer #2:  General comments:  All the issues raised here have been addressed  Title: The title has been modified (L2-3)  Abstract: This has been improved’(L23-42)  This has been addressed  Methods: This has been addressed(L27-30)  Conclusion: This has been modified (L43-45)  Introduction: This has been addressed (L51-82)  This has been addressed  The rationale for the study has been justified  (L75-78) |
|  | Materials and methods:  Line 87: Authors should provide the names of the study institution and the rural community.  Line 97 to 99: Authors should note that an infection identified during a hospital visit will not qualify as asymptomatic.  Line 100: Authors should specify ‘recent history’ (e.g. 24or 48 hours ago or a week ago etc.) in their inclusion criteria/definition of asymptomatic infection  Line 104: Why did Authors exclude individuals with mental illness? Was axillary temperature ≥ 37°C also excluded?  Line 110 to 120: The procedure of the systematic sampling is impressive, however, it is unclear why the sampling is considered random, as the selection of the first respondent appears systematic.  Line 147: May not be relevant.  Line 148, 157: Authors should structure as a minor heading, removing the indent.  Line 161 to 163: Authors should ensure that the referenced treatment guidelines are for the asymptomatic infections (study target population) and not management of clinical malaria. | Materials and methods  The name of the study institution and the rural community have been added (L87-89)  Recent history has been specified (L104-106)  Exclusion criteria has been re-written (L111-113)  This has been addressed(L107-113)  This has been addressed  (L124-125)  Reference treatment guideline has been modified |
|  | Authors should provide a clear definition of asymptomatic infection in the methods section.  Results: The authors aim to demonstrate association between several predictor variables and asymptomatic infections, however, the lack of a clear definition for asymptomatic infection (ASM) detracts from the integrity of the data collected. Specifically,  Line 176: Of respondents who tested positive for parasitaemia, how many had symptoms?  Line 179, Table 1: The proportion of individuals testing negative has not been shown to have asymptomatic infections (by PCR) and so should be removed from Table 1, or the heading re-worded. The individuals testing negative should not be placed under the heading ‘Asymptomatic Malaria Parasitaemia’ in the table.  Line 183 to 203: Table 2, 3 and 4 show the distribution of microscopy positive and negative tests by age, sex, occupation etc… and whether the overall differences in the distribution are significant. The tables do not show association between the predictor variables and asymptomatic infections (ASM). Authors should present their results/data properly, for statistical accuracy.  Line 188, Table 2: The reference variables are unclear, if association between the predictors and ASM is the goal. The negative population may or may not have malaria parasites by PCR, so ‘Malaria’ should be replaced with the word ‘Microscopy’.  Line 216, Table 6: The response variable (malaria test positive with or without symptoms = ASM?) for which the regression analyses was performed is missing from the table, so it is difficult to understand how the results were interpreted by the authors.  Discussion: The discussion reads well, based on the results presented. However, due to the lack of a clear definition for ASM and inaccurate interpretations of the statistical analyses, the discussions and conclusions cannot be scientifically supported. The discussion should be re-written after the corrections in the other sections. | This has been addressed(L178-179)  Results: The results section has been re-written (L194-224)  This has been addressed in Table 1,2,)  This has been addressed in table 3-6  This has been addressed in table 3  Table 6 has been modified/re-analyzed  Discussion:  The whole of the discussion section has been re-written. (L255-304)  END. |
