# Supplementary material for: Prevalence of asymptomatic malaria infection by microscopy and its determinants among residents of Ido-Ekiti, Southwestern Nigeria
Source: PLoS One. 2023 Feb 14;18(2):e0280981. doi: 10.1371/journal.pone.0280981 (PMC9928065; doi:10.1371/journal.pone.0280981)
Supplement: Attachment — Submitted filename: Reviewer.doc [file pone.0280981.s005.doc]

**Summary of reviewer’s comments and Authors’ response (PONE-22-24029R1)**

**Reviewer: 1**

| comments | **Response from Author:** |
| --- | --- |
| Authors have addressed the previous comments well. However, there are minor comments and suggestions to make the manuscript better for the readers and scientific community.  **Minor comments:**  I think it is good to add the word “malaria” in your title (Prevalence of asymptomatic malaria infection..)  **Abstracts:**  The background is still not adequate. You have to show the gap. I suggest writing “Asymptomatic infections have received--- Few epidemiological studies on …However; there is limitation on data regarding asymptomatic infections by microscopy and its determinants among residents of Ido-Ekiti, Southwestern Nigeria”  **Materials and methods:**  Authors have put “ study population” which is good. But, they also have to write” Source population”  Line 185 “Quantitative data are (Change “are” to were)  **Results:**  My previous concern on regression analysis section is not addressed. For example, why did you use different measures of association? In table 3,4, and 5 you used chi-square, But Odds ratio in table 6. In column, you have to write the number of negatives in each category. You only put number of positive and total. You can leave total, but replace it with negatives. That is why the odds ratios you put in table 6 are not right through direct manual calculation. Odds ratio remains true if you put negative column rather than the total making it clear for the readers.  **Discussion:**  **L**ine 257-258: An overall prevalence of asymptomatic P. falciparum infection in this study was 19.0% (95% CI: 14.1%-24.6%), which is similar to—  Line 264: P. falciparum  Line 275: --5.298 times more likely to have….  Line 283: Why do type 2 diabetic individuals harbor asymptomatic P. falciparum infection rather than developing symptoms?  Line 296:….. five times more likely……  General questions:  The species detected in your study area was P. falciparum only. How much accurate were you? Who examined the slides? Is the area known by the presence of this species alone? If not, I expect that there might have been error in species identification in your study.  Finally align all texts to both the left and right sections. I think all the text is aligned to the left only in your document. | Thank you for the efforts put in to review and improve our manuscript.  Line 1: The word “malaria “ has been added to the title.  **Abstract:**  **L**ine 21-47: The background has been improved and has addressed the suggestions.  **Materials and methods**  **L**ine 106-107: Source population has been included.  **L**ine 186: The word “are” has been changed to “were”  **Results:**  This comment has been addressed. Only one measure of association was now used, which are the odds ratios.  **Discussion:**  **L**ine 228-229: This has been addressed.  **L**ine 235: This has been addressed  **L**ine 246: The word “as likely “ has been changed to “more likely”  **L**ine 260-264: The association between type 2 diabetic individuals and asymptomatic has been addressed on page line  **L**ine 271: Five times “as likely” has been changed to “more likely”  There was no error in species identification in this study. P. falciparum has been the only species known to be prevalent in Southwestern Nigeria and this observation has been corroborated by several studies in Southwestern Nigeria. Check references 7,14,35.  The stained slides were taken to the hospital laboratory, where parasitological examinations were made independently by two malaria microscopists, with discrepancies resolved by a senior microscopist who ensured quality control  **END.** |
