# Supplementary material for: Prevalence of asymptomatic malaria infection by microscopy and its determinants among residents of Ido-Ekiti, Southwestern Nigeria
Source: PLoS One. 2023 Feb 14;18(2):e0280981. doi: 10.1371/journal.pone.0280981 (PMC9928065; doi:10.1371/journal.pone.0280981)
Supplement: Attachment — Submitted filename: Reviewers_1.doc [file pone.0280981.s006.doc]

**Summary of reviewer’s comments and Authors’ response (PONE-D-22-24029R2)**

**Reviewer: 1**

| **Comments** | **Response from Author** |
| --- | --- |
| Reviewer #1: Authors have addressed the previous concerns well. But, here are additional points to be addressed and included to make manuscript more plausible before publication. | Thank you for the efforts put in to review and improve our manuscript. |
| Line 1: Asymptomatic Malaria Infections (remove s) | Line1: The letter ‘s’ has been removed from Infections |
| Line 27: Asymptomatic Malaria Infections (remove s) | Line 27: The letter ‘s’ has been removed from Infections |
| Line 60-61: … clinical immunity which is only partially develops … (remove” which is”) | Line 60-61: The word “which is” has been removed |
| Line 70: Also, the use of…. (Remove also and write as “The use of…) | Line 70: The word ’also’ has been removed and is now written as “The use of….. |
| Line 104- 107: **Study population/ Source population:** This comprised all adult individuals who were residents in Ido-Ekiti, and who presented for the annual free health programs organised by the society of family physicians of Nigeria. The source population comprised apparently healthy individuals aged 40 years and above. | Line 104-107: Now written as;  **Source population:** This comprised all adult individuals who were residents in Ido-Ekiti, and who presented for the annual free health programs organised by the society of family physicians of Nigeria.  **Study population**: The study population comprised apparently healthy individuals aged 40 years and above. |
| Line 108-111: Consented apparently healthy individuals …dizziness, joint pain, anorexia and malaise. | Line 109-113: Now written as;  Consented apparently healthy individuals …dizziness, joint pain, anorexia and malaise were included in the study. |
| Line 112-14: Individuals who were …conduct of this study. | Line 113-115: Now written as;  Individuals who were …conduct of this study were excluded. |
| Line 188: Italicize “Plasmodium” | Line 189: Now written as “*Plasmodium*”  **END** |
